# Presynaptic Release-Regulating Sphingosine 1-Phosphate 1/3 Receptors in Cortical Glutamatergic Terminals: Adaptations in EAE Mice and Impact of Therapeutic FTY720

**DOI:** 10.3390/cells12192343

**Published:** 2023-09-24

**Authors:** Alessandra Roggeri, Guendalina Olivero, Cesare Usai, Tim Vanmierlo, Anna Pittaluga

**Affiliations:** 1 Department of Pharmacy (DiFar), University of Genoa, Viale Cembrano 4, 16148 Genoa, Italy; roggeri@difar.unige.it (A.R.); olivero@difar.unige.it (G.O.); 2 Institute of Biophysics, National Research Council, Via De Marini 6, 16149 Genoa, Italy; cesare.usai@ibf.cnr.it; 3 Department of Neuroscience, Biomedical Research Institute, European Graduate School of Neuroscience, Hasselt University, B-3590 Hasselt, Belgium; tim.vanmierlo@uhasselt.be; 4 Department of Psychiatry and Neuropsychology, School for Mental Health and Neurosciences, Division Translational Neuroscience, Maastricht University, 6200 MD Maastricht, The Netherlands; 5 Department of Pharmacy (DiFar), Center of Excellence for Biomedical Research, 3Rs Center, University of Genoa, Viale Cembrano 4, 16148 Genoa, Italy; 6 IRCCS Ospedale Policlinico San Martino, 16145 Genoa, Italy

**Keywords:** synaptosomes, sphingosine-1-phosphate receptor, glutamate, EAE mice, FTY720

## Abstract

This study provides evidence of the existence of presynaptic inhibitory sphingosine-1-phosphate receptor 1 (S1P1R) and facilitatory S1P3R in cortical nerve endings (synaptosomes) of healthy mice. The conclusion relies on the findings that (i) the S1P1R agonist CS-2100 (0.1–30 nM) inhibits the 12 mM KCl-evoked glutamate exocytosis (quantified as the release of [^3^H]D-aspartate) while the S1P3R allosteric agonist CYM-5541 potentiates it and (ii) these effects are inhibited by the S1P1R antagonist Ex 26 (30–300 nM) and the S1P3R antagonist TY-52156 (100–1000 nM), respectively. Confocal microscopy and western blot analysis confirmed the presence of S1P1R and S1P3R proteins in cortical glutamatergic synaptosomes, which were scarcely accessible to biotin in a biotinylation study. Then, we demonstrated that S1P1R and S1P3R densities and their release activity are amplified in cortical synaptosomes of mice suffering from experimental autoimmune encephalomyelitis (EAE), despite receptors maintain their preferential internal distribution. Receptor changes recover following chronic oral therapeutic FTY720 (0.03 mg/Kg/day). These results improve our knowledge of the role of presynaptic release-regulating S1P1Rs and S1P3Rs controlling glutamate transmission in the CNS also unravelling functional adaptations during EAE that recover following chronic FTY720. In a whole, these findings provide new information on the central neuroprotectant activities of FTY720.

## 1. Introduction

Sphingosine-1-phosphate receptors (S1PRs) belong to class A G protein-coupled receptors. They are divided into five different subtypes (S1P1–S1P5 receptors) and couple to different G proteins, triggering either excitatory or inhibitory effects based on associated transduction pathways. They are widely distributed in the central nervous system (CNS), where they are expressed in immune cells, microglia, astrocytes, neurons, and oligodendrocytes. Among the different subtypes, S1P5R has a preferential distribution in oligodendrocytes, while S1P1R and S1P3R subtypes are expressed in astrocytes and neurons [1,2].

Besides the “immunological” role in controlling T lymphocyte egression and trafficking, S1PRs have been proposed to exert central “non -immunological” functions that support the neuroprotective and anti-excitotoxic effects of S1PR ligands [3,4,5,6,7]. The issue is particularly relevant taking into consideration that (i) alteration in synaptic transmission favors the onset of synaptopathy, the main pathogenic mechanism in different central diseases, including multiple sclerosis (MS), [8,9,10] and (ii) drug(s) able to restore the efficiency of synaptic connections can provide new therapeutic approaches for the management of these pathologies. For example, the broad spectrum S1PR modulator fingolimod (FTY720) [11], the first drug approved for oral treatment of MS, is now under validation for the management of other central disorders [7,12,13,14]. When therapeutically administered, FTY720 reduces brain volume loss in patients suffering from relapsing-remitting MS [15]. Furthermore, it counteracts synaptic dysfunctions in mice suffering from experimental autoimmune encephalomyelitis (EAE), returning glutamatergic transmission to physiological levels in selected brain regions, including the cortex, the hippocampus, and the striatum [16,17,18]. Although the “immunological” activity of FTY720 is undoubtedly pivotal to its therapeutic activities, the possibility that its central “non-immunological” therapeutic effects may also involve S1PRs to control synaptic connection is attractive and deserves further investigation. 

The recent literature on S1PRs suggests that these receptors directly control the release of glutamate in neurons [6,19,20,21,22]. In our opinion, these studies need further analysis to deepen our understanding of the role of these receptors as modulators of glutamate exocytosis from nerve terminals and to highlight possible pathological adaptations that might affect their density/activity during demyelinating diseases and that could be selectively targeted by drugs such as FTY720. To answer these questions, our study focused on two S1PR subtypes, S1P1 and S1P3 receptors, which, based on the available literature, are predicted to be located presynaptically in glutamatergic nerve endings (synaptosomes) and control glutamate exocytosis [20,21,23]. We first characterized these receptors in the cortex of healthy mice from a functional and biochemical point of view. We then analyzed their density and functions in EAE mice and investigated whether their adaptations could be targeted by FTY720.

## 2. Materials and Methods

### 2.1. Animals

Mice (female, strain C57BL/6J) were purchased from Charles River (Calco, Italy) and housed in the animal facility at the Department of Pharmacy, School of Medical and Pharmaceutical Sciences, University of Genoa (authorization n. 484 of 8 June 2004). EAE mice were sacrificed 25 ± 1 d.p.i. via cervical dislocation and then decapitated to collect the cortices. The experimental procedures were conducted in accordance with the European legislation (Directive 2010/63/EU for animal experiments) and the ARRIVE guidelines. They were approved by the Animal Subjects Review Board of the University of Genoa and by the Italian Ministry of Health (DDL 26/2014 and previous legislation; protocol no. 50/2011-B and 612/2015-PR).

### 2.2. EAE Induction and Clinical Scores

Mice (female, strain C57BL/6J, 18–20 g, 6–8 weeks) were immunized following a standard protocol, with minor modifications [24]. Briefly, animals were administered incomplete Freund’s adjuvant containing 4 mg/mL Mycobacterium tuberculosis (strain H37Ra) and 200 μg of the myelin oligodendrocyte protein 35–55 (MOG_35–55_). This was followed by intraperitoneal administration of 400 ng of Pertussis toxin on the day of immunization and two days after. Animals were scored daily for clinical symptoms of EAE according to the following scale: 0, no clinical signs; 1, flaccid tail; 2, hindlimb weakness; 3, hindlimb paresis; 4, tetraparalysis; 5, death; intermediate clinical signs were scored by adding 0.5 [17]. 

### 2.3. Drug Treatment

FTY720 was administered orally—dissolved in drinking water (0.03 mg/Kg)—following an already validated therapeutic protocol [17]. Drug administration started 11 ± 1 d.p.i. and continued for 14 days.

### 2.4. Synaptosomes 

Purified synaptosomes were prepared as previously described [25]. Briefly, after decapitation, the cortices were rapidly removed and homogenized in 0.32 M sucrose, buffered to pH 7.4 with Tris-(hydroxymethyl)-amino methane [Tris, final concentration (f.c.) 0.01 M] using a glass/Teflon tissue grinder (clearance 0.25 mm). The homogenates were centrifuged at 1000× *g* for 5 min to remove the nuclei and cellular debris and the supernatant was stratified on a discontinuous Percoll^®^ gradient (2%, 6%, 10%, and 20% *v*/*v* in Tris-buffered sucrose) and centrifuged at 33,500× *g* for 6 min. The layer between 10% and 20% Percoll^®^ (synaptosomal fraction) was collected and washed by centrifugation.

### 2.5. Superfusion Experiments 

Synaptosomes were resuspended in a physiological medium with the following composition (mM): NaCl, 140; KCl, 3; MgSO_4_, 1.2; CaCl_2_, 1.2; NaH_2_PO_4_, 1.2; NaHCO_3_, 5; HEPES, 10; glucose, 10; pH 7.2–7.4, and incubated for 15 min at 37 °C in a rotary water bath in the presence of [^3^H]D-aspartate (f.c. 50 nM), an unmetabolizable analog of glutamate that efficiently mimics the uptake, the vesicular storage, and the exocytosis of glutamate from synaptosomes [26]. Identical portions of the synaptosomal suspensions were stratified on microporous filters at the bottom of parallel chambers in a Superfusion System (Ugo Basile, Comerio, Varese, Italy) [27] and maintained at 37 °C. The particles were superfused with a standard physiological solution at 0.5 mL/min. After 39 min of superfusion to equilibrate the system, synaptosomes were transiently exposed (90 s) to a high KCl-containing solution (12 mM KCl solution; NaCl substituting for an equimolar concentration of KCl) in the presence or absence of S1PR agonists [sphingosine-1-phosphate (S1P), CS-2100, and CYM-5541] or antagonists [Ex 26 and TY-52156]. When not otherwise indicated, the superfusion was carried out under resting conditions (in a medium containing 3 mM KCl). Three superfusate fractions (two basal 3-min fractions, one before (t = 36–39 min; fraction 1) and one after (t = 45–48 min; fraction 3) a 6-min fraction (t = 39–45 min; fraction 2)) were collected. These fractions and the filters (containing the superfused particles) were counted for radioactivity. The amount of radioactivity released into each fraction was expressed as a percentage of the total radioactivity (i.e., the sum of radioactivity in the collected fractions and the superfused synaptosomes at the end of the experiments). The KCl-induced overflow was estimated by subtracting the neurotransmitter content collected from the first and third fractions from that collected from the 6-min fraction (fraction 2). The effect of the compounds was expressed as a percentage of the respective tritium overflow elicited by the KCl solutions (3 mM and 12 mM).

### 2.6. Confocal Microscopy

Mouse cortical synaptosomes (40 μg of proteins) were fixed with 2% paraformaldehyde, permeabilized with 0.05% Triton X-100 PBS and incubated in suspension overnight at 4 °C with the following primary antibodies diluted in 3% bovine serum albumin (BSA) in PBS: rabbit anti-S1P1R (1:600, AB11424, Abcam, Cambridge, UK), rabbit anti-S1P3R (1:300, AB108370, Abcam, Cambridge, UK), goat anti-syntaxin 1A (1:3000, AF7237, R&D systems, Minneapolis, MN, USA), and guinea pig anti-vesicular glutamate transporter type 1 (VGLUT1; 1:1000, AB5905, Millipore, Darmstadt, Germany). Particles were then washed in PBS and incubated for 1 h at room temperature with the respective secondary antibodies diluted in 3% BSA/PBS: donkey anti-rabbit AlexaFluor-555 (1:900, A-31572, Life Technologies; Thermo Fisher Scientific, Waltham, MA, USA), donkey anti-guinea pig AlexaFluor-488 (1:900, AB_2340472, Jackson ImmunoReasearch, Pennsylvania, PA, USA), and donkey anti-goat AlexaFluor-647 (1:900, A-21447, Life Technologies). Synaptosomes were resuspended in ProLong™ Gold Antifade Mountant (P10144; Life Technologies) and then applied onto coverslips. Fluorescence images (512 × 512 × 8 bit) were acquired using a three-channel Leica TCS SP2 laser-scanning confocal microscope equipped with 458, 476, 488, 514, 543, and 633 nm excitation lines through a plan-apochromatic oil immersion objective 63×/1.4 NA. Based on the objective properties, images were approximately 246 × 246 μm^2^ in area. Light collection configuration was optimized based on the combination of chosen fluorochromes, and sequential channel acquisition was performed to avoid crosstalk. Background subtraction was performed whenever necessary. The Leica ‘LAS AF’ software package was used for image acquisition. Quantitative estimation of co-localized proteins was performed by calculating the Pearson correlation coefficient (P), expressing the spatial correlation between two channels of a dual-color image, and calculating Mander’s colocalization coefficient (M) [28]. The fraction of co-localizing molecular species in each component of a dual-color image was expressed; if two molecular species are co-localized, the overlay of their spatial distributions has a correlation value higher than what would be expected by chance alone. In 2004, Costes and colleagues [29] developed an automated procedure for evaluating the correlation between the green and red channels with a significance level > 95%. The same procedure automatically determines an intensity threshold for each color channel based on a linear least-square fit of the green and red intensities in the image’s 2D correlation cytofluorogram. Costes’ approach was conducted using macro routines integrated as plugins (WCIF Colocalization Plugins, Wright Cell Imaging Facility, Toronto Western Research Institute, Toronto, ON, Canada) in the ImageJ 1.53q software (Wayne Rasband, NIH, USA). 

### 2.7. Western Blot Analysis

Cortical synaptosomes of mice from the different experimental groups (control, FTY720-untreated EAE, and FTY720-treated EAE) were lysed in modified RIPA buffer (10 mM Tris, pH 7.4; 150 mM NaCl; 1 mM EDTA; 0.1% SDS; 1% Triton X-100; protease inhibitors) and the protein content was determined using the Pierce™ BCA assay kit (23225, Thermo Scientific, Waltham, MA, USA). Samples were resuspended in SDS-PAGE loading buffer, boiled at 95 °C for 5 min, separated on 7.5% SDS-PAGE (20 μg/lane), and transferred onto polyvinylidene difluoride (PVDF) membranes (GE10600023, Amersham). Membranes were incubated for 1 h at room temperature in Tris-buffered saline-Tween (t-TBS: 0.02 M Tris, 0.15 M NaCl, and 0.05% Tween 20) containing 5% (*w*/*v*) non-fat dried milk and then incubated overnight at 4 °C with different primary antibodies: rabbit anti- CD11b (1:2500, AB13357, Abcam, Cambridge, UK), rabbit anti-MAP2 (1:2000, GTX133109, GeneTex, Irvine, CA, USA), mouse anti-GFAP (1:5000, G3893, Sigma-Aldrich, St. Louis, MO, USA), rabbit anti-GluA2 (1:2000, AB206293, Abcam, Cambridge, UK), rabbit anti-S1P1R (1:1000, AB11424, Abcam, Cambridge, UK), rabbit anti-S1P3R (1:300, AB108370, Abcam, Cambridge, UK), and mouse anti-β tubulin (1:500, T8660, Merck Life Science, Milan, Italy). After three washes in t-TBS (0.05%), membranes were incubated for 1 h at room temperature with appropriate horseradish peroxidase-linked secondary antibodies (1:10,000, A9044 (anti-mouse) and A9169 (anti-rabbit), Sigma-Aldrich, St. Louis, MO, USA). Immunoblots were visualized using the enhanced chemiluminescence western blotting detection system Immobilon Forte Western HRP substrate (WBLUF0100, Merck Life Science, Milan, Italy). Images were acquired using the Alliance LD6 image capture system (Uvitec, Cambridge, UK) and analyzed using the UVI-1D software.

### 2.8. Biotinylation Studies

Mouse cortical synaptosomes were divided into two aliquots. The first aliquot was lysed in modified RIPA buffer and used to analyze S1P1R and S1P3R protein contents in the total synaptosomal lysate (L). The other aliquot was labeled with EZ-Link Sulfo-NHS-SS-Biotin (2 mg/mL; 21,331 Thermo Scientific) for 1 h at 4 °C in PBS/Ca^2+^-Mg^2+^ with the following composition (mM): 138 NaCl, 2.7 KCl, 1.8 KH_2_PO_4_, 10 Na_2_HPO_4_, 1.5 MgCl_2_, 0.2 CaCl_2_, pH 7.4. The biotinylation reaction was stopped by incubating synaptosomes with PBS/Ca^2+^-Mg^2+^ containing 100 mM glycine for 20 min at 4 °C. After two washes in PBS/Ca^2+^-Mg^2+^, synaptosomes were lysed in modified RIPA buffer. The biotinylated samples (200 µg) were incubated, under shaking, with Dynabeads MyOne Streptavidin T1 beads (65601, Invitrogen) for 30 min at room temperature to pulldown the biotinylated proteins. Non-biotinylated samples (200 µg) were analyzed following the same procedure to check the specificity of streptavidin pulldown (negative control, N). After three washes in PBS-Tween (0.01%), the samples were resuspended in SDS-PAGE loading buffer and boiled for 5 min at 95 °C to separate biotinylated proteins from the beads. The beads were discarded and the supernatant was loaded on a polyacrylamide gel (7.5%) and analyzed using an immunoblot assay. The immunoreactivity of S1P1/3Rs was detected using rabbit anti-S1P1R (1:1000, AB11424, Abcam, Cambridge, UK) and rabbit anti-S1P3R (1:300, AB108370, Abcam, Cambridge, UK) antibodies in the total synaptosomal lysate (L), in streptavidin pulldown of the non-biotinylated synaptosomal lysate (N), and in the biotinylated synaptosomes samples (B). Mouse anti-β tubulin (1:500, T8660, Sigma, St. Louis, MO, USA) and rabbit anti-GluA2 (1:2000, AB206293, Abcam, Cambridge, UK) were used as negative and positive controls, respectively, in the biotinylation protocol.

### 2.9. Statistical Analysis

**Figure 1:**
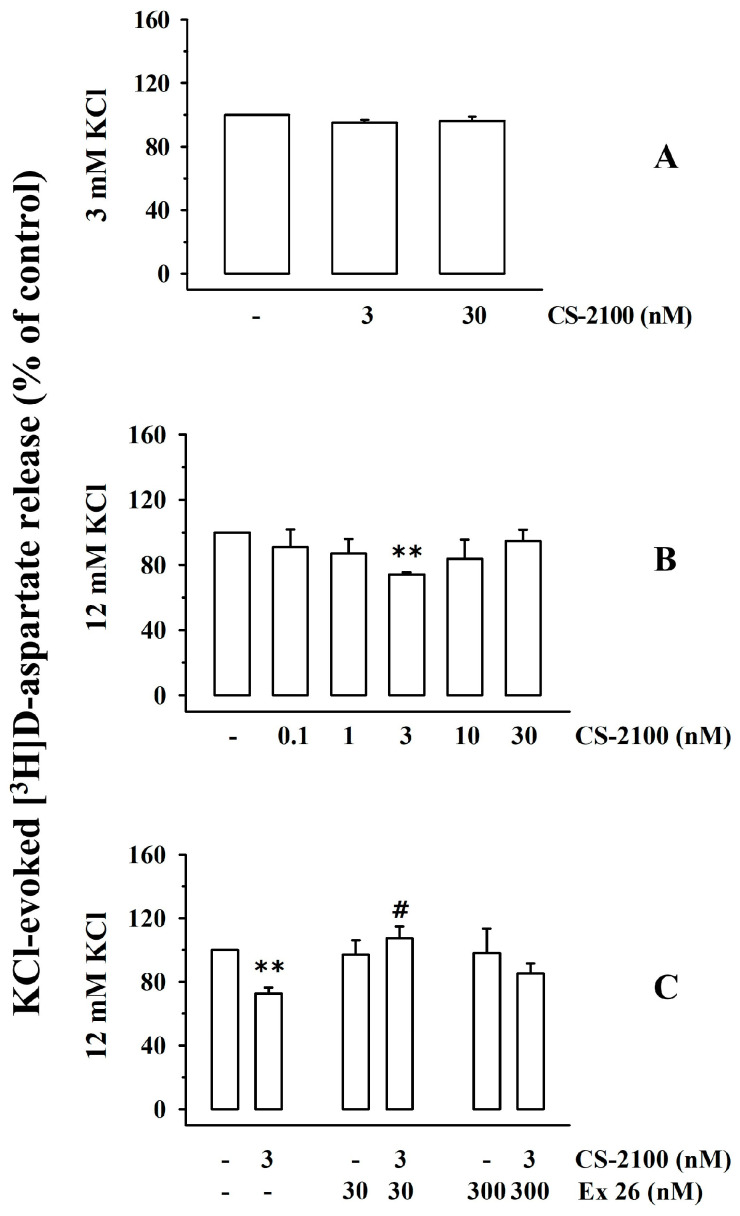
Effects of the S1P1R agonist, CS-2100, on the spontaneous and KCl-evoked release of [^3^H]D-aspartate from mouse cortical synaptosomes: reversal by the S1P1R antagonist Ex 26. Synaptosomes from the cortex of healthy mice were preloaded with [^3^H]D-aspartate and then superfused under resting (3 mM KCl, (**A**)) or depolarizing (12 mM KCl, (**B**,**C**)) conditions. Results are expressed as the percentages of the respective controls (the spontaneous (**A**) and KCl-evoked (**B**,**C**) tritium release in the absence of the ligands). Data are means ± SEM of at least five experiments run in triplicate; ** *p* < 0.01 vs. 12 mM KCl-evoked tritium overflow; ^#^ *p* < 0.05 vs. 12 mM KCl/3 nM CS-2100 (3 nM)-evoked tritium overflow.

**Figure 2:**
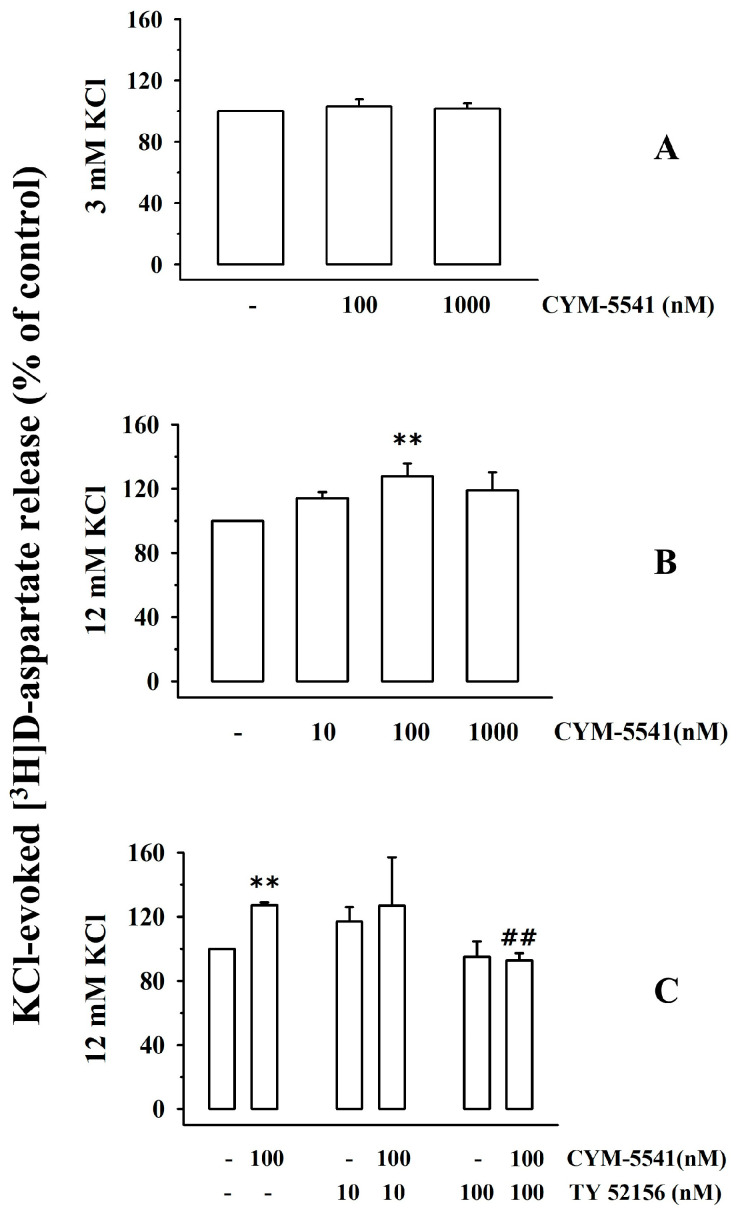
Effect of the S1P3R agonist, CYM-5541, on the spontaneous and KCl-evoked release of [^3^H]D-aspartate from mouse cortical synaptosomes. Reversal by the S1P3R antagonist, TY-52156. Synaptosomes from the cortex of healthy mice were preloaded with [^3^H]D-aspartate and then superfused under resting (3 mM KCl, (**A**)) or depolarizing (12 mM KCl, (**B**,**C**)) conditions. Results are expressed as percentages of the respective controls (the spontaneous (**A**) and KCl-evoked (**B**,**C**) tritium release in the absence of ligands). Data are means ± SEM of at least five experiments run in triplicate; ** *p* < 0.01 vs. 12 mM KCl-evoked tritium overflow; ^##^ *p* < 0.01 vs. 12 mM KCl/100 nM TY-52156-evoked tritium overflow.

SigmaPlot data analysis and graphing software package (10.0 version) was used for data handling/analysis and for drawing graphs. Analysis of variance was performed using ANOVA followed by Dunnett’s test (Figures 1A,B and 2A,B) or Tukey–Kramer (8) multiple-comparisons test. Direct comparisons were performed using Student’s *t*-test (Figures 1C, 2C, 8 and 9). Post-hoc tests were performed only if the F value was significant. Results were considered significant at *p* < 0.05.

### 2.10. Drugs and Chemicals

Aspartic acid, D-[2,3-3H] was obtained from Perkin Elmer (NET581001MC, Boston, MA, USA). Pertussis toxin was acquired from List Biological Laboratories (#181, Campbell, Santa Clara Country, California, USA) and incomplete Freund’s adjuvant was obtained from Sigma-Aldrich (F5506, Saint Louis, MO, USA). Myelin oligodendrocyte glycoprotein (MOG) was purchased from Espikem (EPK1, Florence, Italy). Mycobacterium tuberculosis (H37Ra) was obtained from DIFCO BACTO Microbiology (241141, Becton, MD, USA). FTY720 was supplied by Novartis Pharma AG (Basel, Switzerland). N,N-dimethylsphingosine (DMS, 4640), sphingosine-1-phosphate (S1P, 1370), CS-2100 (4543, EC50 = 4.0 nM), CYM-5541 (4897, EC50 = 72–132 nM), Ex 26 (5833, IC50 = 0.93 nM), and TY-52156 (5328, Ki = 110 nM) were all purchased from Tocris Bioscience (Bristol, UK).

## 3. Results

### 3.1. Presynaptic Release-Regulating S1P1 Receptors in Cortical Synaptosomes of Healthy Mice

In 2021, Wang and colleagues proposed the existence of presynaptic release-regulating S1P1Rs in cortical glutamatergic nerve endings [21]. To implement the pharmacological characterization of these receptors, release experiments were conducted to investigate the impact of the S1P1R agonist, CS-2100 [30], and the S1P1R antagonist, Ex 26 [31], on the release of glutamate (monitored as the release of preloaded [^3^H]D-aspartate [26]) from cortical synaptosomes of healthy mice. First, we investigated the efficiency of the S1P1R agonist in modifying the spontaneous release of glutamate (quantified as release of [^3^H]D-aspartate, Figure 1A) and then we verified its efficiency in controlling the KCl-evoked release of tritium from cortical synaptosomes (Figure 1B). 

CS-2100 (3–30 nM) did not modify the release of [^3^H]D-aspartate under resting conditions (i.e., 3 mM KCl, Figure 1A), but reduced the 12 mM KCl-evoked [^3^H]D-aspartate exocytosis from cortical synaptosomes (Figure 1B). The agonist inhibited the tritium overflow in a concentration-dependent manner (0.1–30 nM) and was maximally active at 3 nM. 

To confirm—from a pharmacological point of view—the involvement of the presynaptic S1P1Rs in the releasing activity, experiments were carried out to verify whether the S1P1R antagonist, Ex 26, can antagonize the inhibitory effect elicited by CS-2100. The S1P1R antagonist, Ex 26 (30–300 nM), did not affect the tritium overflow elicited by the high KCl solution (Figure 1C) but largely reverted the 3 nM CS-2100-induced inhibition of the 12 mM KCl-evoked [^3^H]D-aspartate exocytosis when concomitantly added to the agonist (Figure 1C). These results confirm—from a pharmacological point of view—that the presynaptic inhibitor S1PR involved in the CS-2100-induced effect belongs to subtype 1. We concluded that cortical synaptosomes are endowed with presynaptic release-regulating S1P1Rs whose activation does not modify the spontaneous release of glutamate but inhibits the exocytosis elicited by a mild depolarizing stimulus.

### 3.2. Presynaptic Release-Regulating S1P3 Receptors Exist in Cortical Synaptosomes of Healthy Mice

**Figure 3:**
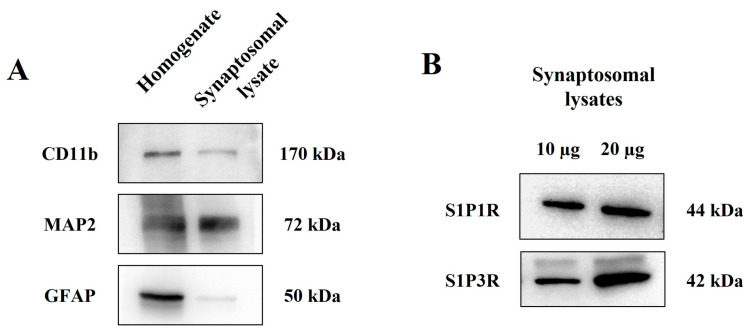
Cortical synaptosomes are endowed with S1P1R and S1P3R. (**A**) Comparative analysis of the presence of astrocytes (GFAP-immunopositivity), microglia (CD11b-immunopositivity), and neuron (MAP2 immunopositivity) markers in total cortical homogenates and in synaptosomal lysates. (**B**) Western blot analysis of S1P1R and S1P3R proteins in cortical synaptosomal lysates. The figure shows representative images of three (**A**) and four (**B**) different analyses carried out on different samples on different days.

Experiments were then carried out to verify the existence of S1P3R. We focused on this receptor subtype based on some conflicting observations in the literature concerning its potential role as a presynaptic release-regulating receptor (see the discussion, [20, 21]. First, release experiments were carried out to study the impact of the S1P3R-positive allosteric modulator, CYM-5541, on the spontaneous and KCl-evoked release of tritium from cortical synaptosome. CYM-5541 (100–1000) did not modify the release of tritium under resting conditions (3 mM KCl, Figure 2A), but potentiated the 12 mM KCl-evoked [^3^H]D-aspartate overflow in a concentration-dependent (10–1000 nM) manner (Figure 2B). The maximum effect of CYM-5541 was observed when 100 nM of the ligand was added (Figure 3B). CYM-5541-evoked tritium overflow was inhibited by the concomitant addition of the S1P3R antagonist, TY-52156 (100 nM, Figure 2C [32]). The antagonist (0.1–1 µM) did not modify the 12 mM KCl-evoked release of [^3^H]D-aspartate on its own (Figure 2C). 

The functional observations support the conclusion that cortical glutamatergic nerve endings are also endowed with presynaptic facilitatory release-regulating sphingosine receptors with a pharmacological profile consistent with the S1P3R subtype that positively controls the exocytosis of [^3^H]D-aspartate, leaving its spontaneous outflow unchanged. 

### 3.3. Cortical Glutamatergic Synaptosomes of Healthy Mice Are Endowed with S1P1 and S1P3 Receptor Proteins

To further support the existence of S1P1 and S1P3 autoreceptors on cortical glutamatergic synaptosomes, western blot studies, and confocal analyses were performed to verify the presence of S1P1R and S1P3R proteins in these particles. Preliminary experiments were dedicated to verifying the purity of the synaptosomal fraction, which was assessed using antibodies against astrocyte (glial fibrillary acidic protein, GFAP), neuron (microtubule-associated protein 2, MAP2), and microglia (CD11b protein) markers to highlight the presence of glial and astrocytic-derived particles in the synaptosomal preparation. The immunopositivity for the three markers was concomitantly analyzed in cortical homogenate and synaptosomal lysate. The three proteins were largely expressed in the cortical homogenate. Conversely, the synaptosomal lysates were immunopositive for MAP2, whereas GFAP and CD11b immunostainings were scarce, almost undetectable (Figure 3A). These results indicate negligible contamination of astrocytes and glial components in the synaptosomal fraction and support the conclusion that the cortical preparation mainly consists of isolated nerve endings (i.e., synaptosomes).

The synaptosomal preparation was then analyzed for the presence of S1P1 and S1P3 receptor proteins. The S1P1R antibody highlighted the presence of an immunostain consistent with the mass of the S1P1R protein in cortical synaptosomal lysates (10 and 20 µg) (44 kDa, Figure 3B). Similarly, the anti-S1P3R antibody staining showed the presence of immunopositivity with a mass (42 kDa) corresponding to the S1P3R protein (Figure 3B). 

**Figure 4:**
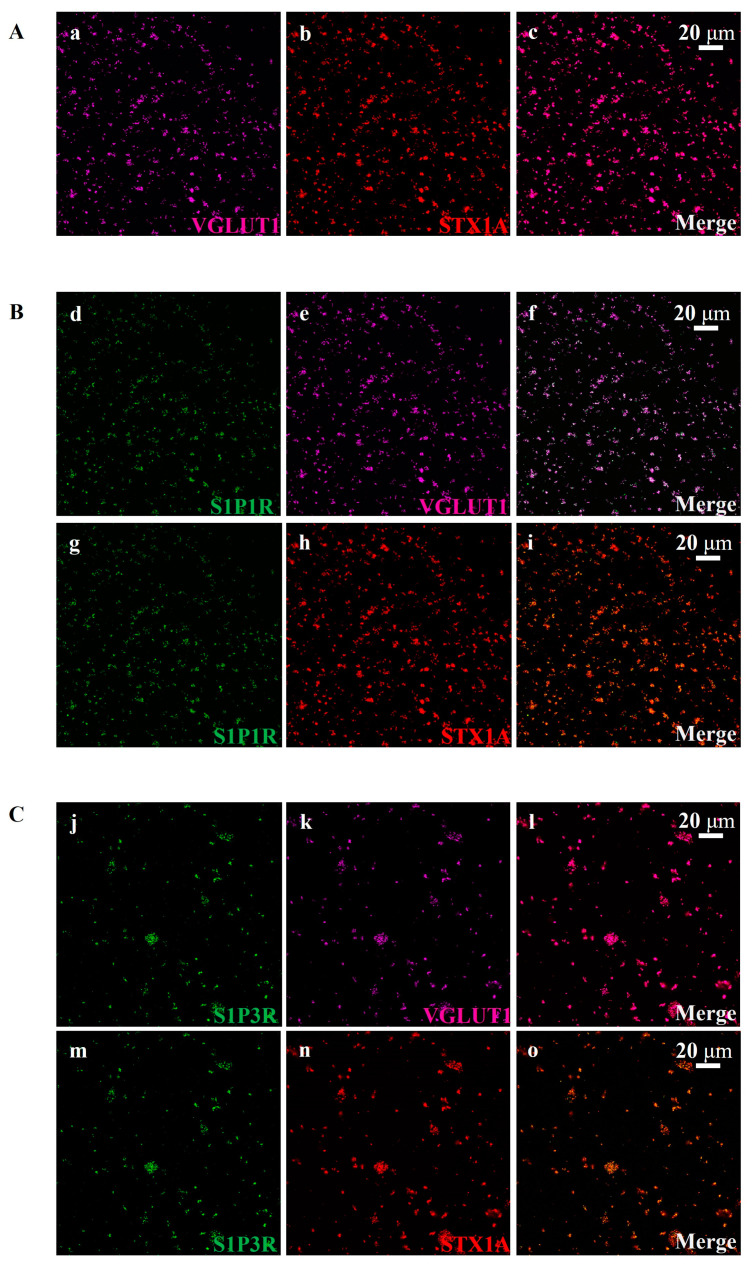
S1P1R and S1P3R located on glutamatergic nerve terminals isolated from the cortex of control mice. (**A**) Colocalization (**c**, merge, purple) of vesicular glutamate transporter type 1 (VGLUT1, **a** magenta) and syntaxin-1a (STX1A, **b**, red). (**B**) Colocalization (**f**, merge, pink) of S1P1R (**d**, green) and VGLUT1 (**e**, magenta) and colocalization (**i**, merge, yellow) of S1P1R (**g**, green) and STX1A (**h**, red) in mouse cortical synaptosomes. (**C**) Colocalization (**l**, merge, pink) of S1P3R (**j**, green) and VGLUT1 (**k**, magenta) and colocalization (**o**, merge, yellow) of S1P3R (**m**, green) and STX1A (**n**, red) in mouse cortical synaptosomes. The figure shows representative images of at least five independent experiments carried out on different samples.

The presence of S1P1R and S1P3R in cortical glutamatergic nerve endings was confirmed using confocal microscopy (Figure 4B,C, respectively). This technique enables the discrimination of different synaptosomal subpopulations (i.e., glutamatergic, GABAergic, etc.) and evidentiates the presence of the receptor proteins in a selected subpopulation. In particular, glutamatergic synaptosomes were identified using antibodies against vesicular glutamate transporter type 1 (VGLUT1, Figure 4(Aa), magenta) and syntaxin-1A (STX1A) protein (Figure 4(Ab), red). VGLUT1 and STX1A were efficiently stained in the mouse cortical synaptosomal preparations (Figure 4(Ab), red) [33]. Merging these images revealed that a high percentage of STX1A-positive particles were also positive for VGLUT1 protein (Figure 4(Ac), merge, purple; Mander’s coefficient: 90.13 ± 4.88%, Pearson correlation coefficient: 0.75 ± 0.07%). 

We then detected a diffuse colocalization (Figure 4(Bf), merge, pink; Mander’s coefficient: 76.17 ± 4.75%, Pearson correlation coefficient: 0.87 ± 0.03%) of S1P1R immunopositivity (Figure 4(Bd,Bg), green) in VGLUT1-positive (Figure 4(Be), magenta) particles and a comparable colocalization (Figure 4(Bi), merge, yellow; Mander’s coefficient: 76.00 ± 4.10%; Pearson correlation coefficient: 0.86 ± 0.02%) in STX1A-positive (Figure 4(Bh), red) particles. S1P3R immunopositivity (green, Figure 4(Cj)) was detected in nerve terminals specialized for glutamate exocytosis, i.e., in STX1A-positive particles (Figure 4(Cn), red; Figure 4(Co), merge, yellow; Mander’s coefficient: 56.33 ± 3.39%; Pearson correlation coefficient: 0.86 ± 0.03%) and in VGLUT1-positive particles (Figure 4(Ck), magenta; Figure 4(Cl), pink, merge; Mander’s coefficient: 58.17 ± 3.00%; Pearson correlation coefficient: 0.87 ± 0.05%). 

### 3.4. Impact of Sphingosine-1-Phosphate and N,N-Dimethylsphingosine on the Release of [^3^H]D-Aspartate from Cortical Synaptosomes of Healthy Mice 

**Figure 5:**
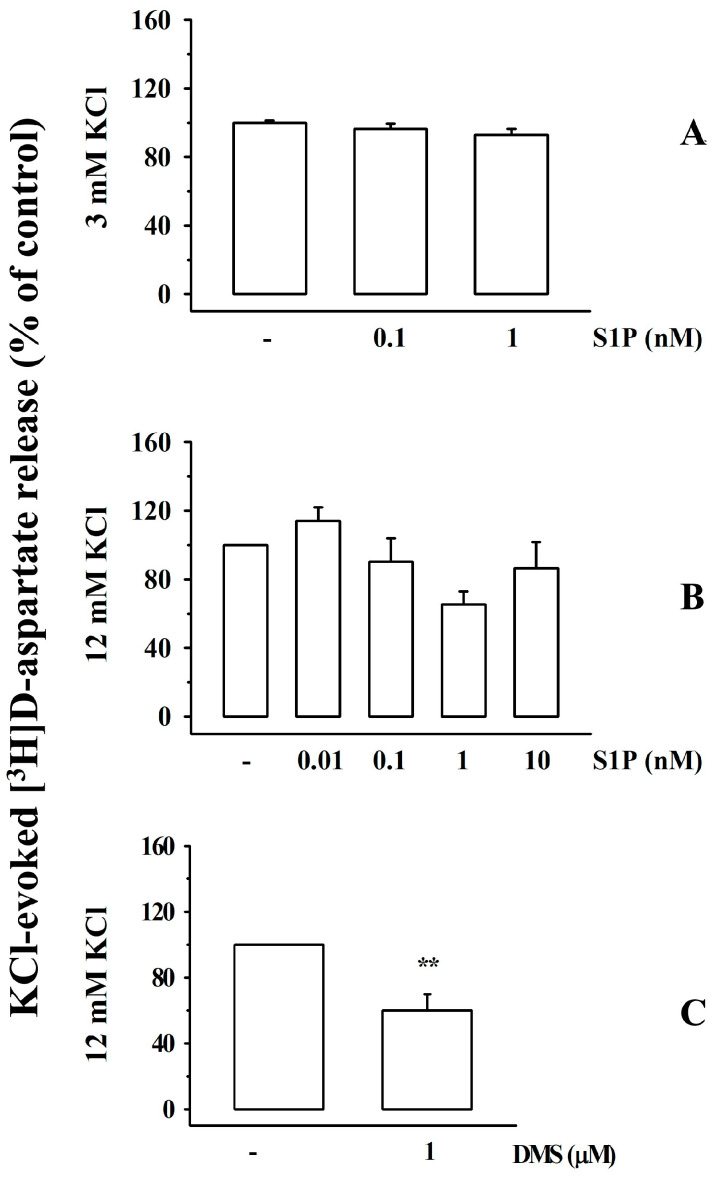
Effects of sphingosine-1-phosphate (S1P) and N,N-dimethylsphingosine on the spontaneous and KCl-evoked release of [^3^H]D-aspartate from mouse cortical synaptosomes. (**A**) Effect of S1P on the [^3^H]D-aspartate release under resting condition or (**B**) elicited by high (12 mM) KCl solution. (**C**) Effect of N,N-dimethylsphingosine (DMS) on the tritium release elicited by high (12 mM) KCl. Results are expressed as a percentage of the respective control. Data are means ± SEM of at least five experiments run in triplicate. ** *p* < 0.01 vs. 12 mM KCl-evoked tritium overflow.

The impact of the broad-spectrum S1PR agonist, sphingosin-1-phosphate (S1P), on [^3^H]D-aspartate release was also investigated. S1P (0.01–10 nM) did not significantly modify the release of [^3^H]D-aspartate under resting conditions (3 mM KCl, Figure 5A) nor did it affect the tritium overflow elicited by transient exposure of synaptosomes to depolarizing stimulus (12 mM KCl solution; Figure 5B). 

Interestingly, the sphingosine kinase (SphK) inhibitor N,N-dimethylsphingosine (DMS, 1 µM) concomitantly added to the high KCl solution significantly reduced the 12 mM KCl-evoked [^3^H]D-aspartate exocytosis (Figure 5C). 

### 3.5. S1P1 and S1P3 Receptors in Cortical Synaptosomal Plasma Membranes: Biotinylation Studies

**Figure 6:**
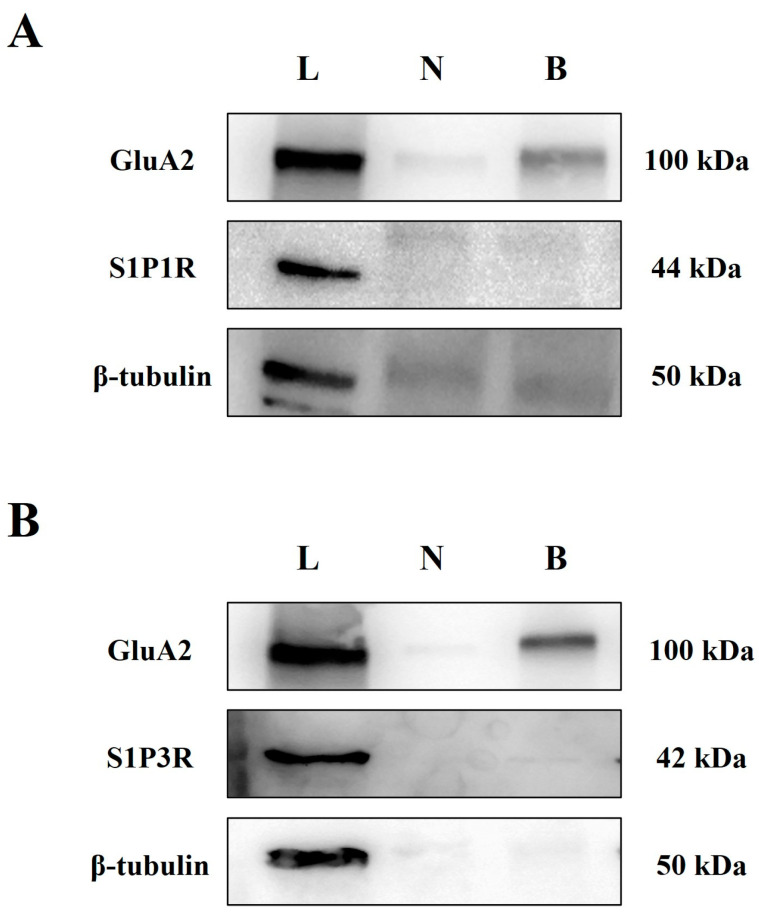
Surface expression of S1P1R and S1P3R proteins on plasma membranes of cortical synaptosomes: biotinylation studies. (**A**) Representative Western blot analysis of the density of the S1P1R and S1P3R (**B**) receptor proteins in cortical synaptosomal plasma membranes. GluA2 protein and β-tubulin proteins were used as positive and negative controls, respectively. The blot compares the total synaptosomal lysate (L), the synaptosomal membranes of cortical synaptosomes that were not exposed to biotin but were subjected to streptavidin pulldown (negative control, N), and the synaptosomal membranes of synaptosomes that were incubated with biotin and then subjected to streptavidin pulldown (biotinylated, B). The blot is representative of three experiments run on different days with different samples.

It has been proposed that S1PRs are expressed in plasma membranes under resting conditions and undergo rapid internalization upon activation with agonists. Biotinylation studies were carried out to verify the presence of S1P1R and S1P3R in the plasma membranes of cortical synaptosomes. Based on previous observations [34], the GluA2 subunit of the AMPA receptors was used as a positive control of the biotinylation process while the β-tubulin was used as a negative control. Based on expectations, GluA2 immunopositivity was detected in the total synaptosomal lysate as well as in the biotinylated fraction (Figure 6A,B), while β-tubulin immunopositivity was absent in the biotinylated samples but present in the synaptosomal lysates (Figure 6A,B). S1P1R and S1P3R immunostainings were detected in synaptosomal lysates but not in the biotinylated samples (Figure 6A,B, respectively). 

### 3.6. Clinical Signs in EAE Mice: Impact of FTY720

**Figure 7:**
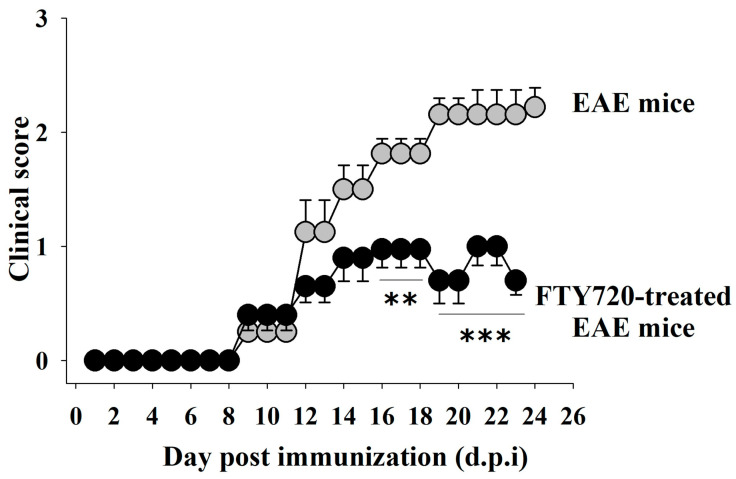
Impact of in vivo therapeutic FTY720 administration on the clinical score of mice with experimental autoimmune encephalomyelitis (EAE). The animal score was recorded daily in fingolimod (FTY720)-untreated (grey circles, *n* = 10) and FTY720-treated EAE mice (black circles, *n* = 10). FTY720 (0.03 mg/kg) (treatment started 11 ± 1 d.p.i., see arrow). The daily clinical score is reported as mean ± SEM; ** *p* < 0.01 vs. FTY720-untreated EAE mice; *** *p* < 0.001 vs. FTY720-untreated EAE mice.

EAE (immunized, MOG_35–55_(+)) female C57BL/6J mice were randomly assigned to the following two groups: FTY720-untreated EAE mice and FTY720 (0.03 mg/Kg)-treated EAE mice. FYT720 was dissolved in drinking water and administered orally following an already validated therapeutic protocol starting from the onset of the first clinical signs [11 ± 1 day post-immunization (d.p.i), see [17] for 14 days. In untreated EAE mice, the severity of the clinical symptoms increased over time and peaked around 19 d.p.i., when the chronic phase was reached (Figure 7, clinical score 16 d.p.i. = 1.8 ± 0.13; clinical score 19 d.p.i = 2.15 ± 0.14). In FTY720-treated EAE mice, the clinical score was significantly lower starting 16 d.p.i. (clinical score 16 d.p.i. = 0.97 ± 0.16, Figure 7) and decreased further 19 d.p.i, (clinical score 19 d.p.i. = 0.70 ± 0.20, Figure 7), consistent with the beneficial effects of the drug on the progression of the disease.

### 3.7. Presynaptic Release-Regulating S1P1 and S1P3 Receptors in Cortical Synaptosomes of EAE Mice Undergo Biochemical and Functional Adaptations That Are Prevented by Therapeutic Chronic FTY720

**Figure 8:**
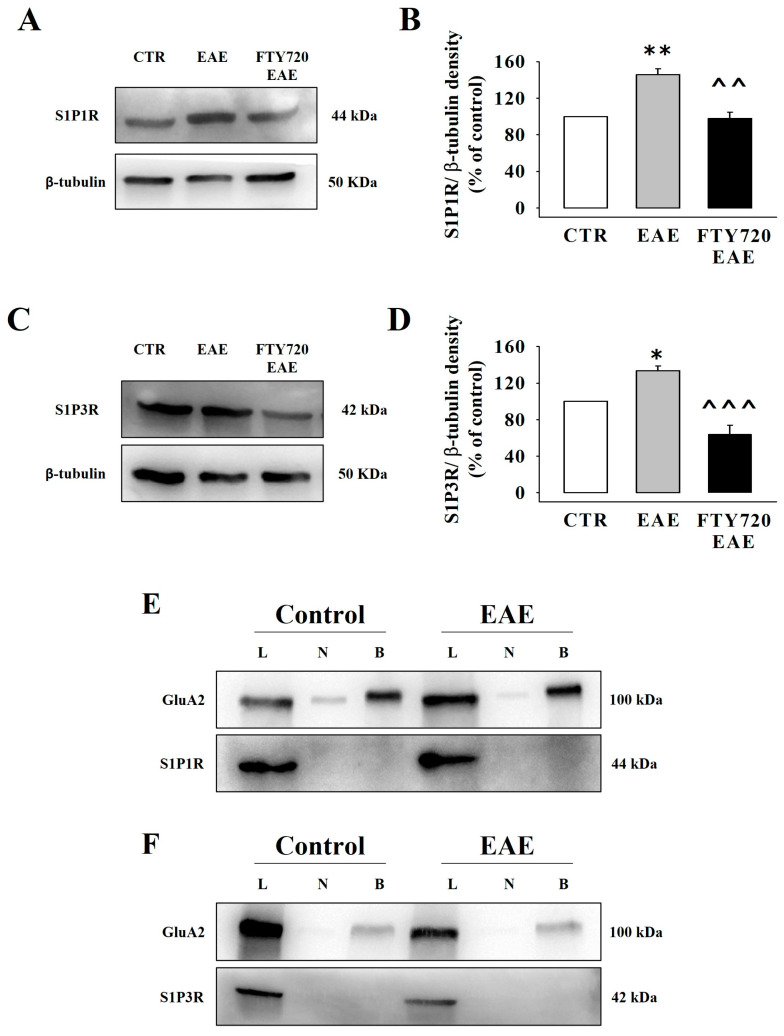
S1P1R and S1P3R densities and surface expressions in cortical synaptosomes of EAE mice at the acute stage of the disease: impact of therapeutic FTY720. (**A**–**D**) The figures show images from representative Western blot analyses of S1P1R (**A**) and S1P3R (**C**) in cortical synaptosomal lysates from control mice, FTY720-untreated EAE mice, and FTY720-treated EAE mice. The blot images are representative of at least five analyses carried out using samples from different immunizations. The densities of S1P1Rs and S1P3Rs were expressed as the S1PR1/β-tubulin ratio (**B**) and the S1PR3/β-tubulin ratio (**D**) and data (mean ± S.E.M.) are reported as percentages of the respective controls; * *p* < 0.05 vs. control mice; ** *p* < 0.01 vs. control mice; ^^^^ *p* < 0.01 vs. FTY720-untreated EAE mice; ^^^^^ *p* < 0.001 vs. FTY720-untreated EAE mice. (**E**,**F**) The figures show images from representative Western blot analyses of biotinylation studies of S1P1R (**E**) and S1P3R (**F**) receptor proteins in cortical synaptosomal plasma membranes. The blot compares the total synaptosomal lysate (L), the synaptosomal membranes of cortical synaptosomes that were not exposed to biotin but were subjected to streptavidin pulldown (negative control, N), and the synaptosomal membranes of synaptosomes that were incubated with biotin and then subjected to streptavidin pulldown (biotinylated, B). The blot is representative of four experiments run on different days with different samples.

We investigated whether MOG-immunization could modify the density and release activity of the presynaptic S1P1Rs and S1P3Rs in cortical synaptosomes and, in the positive, whether chronic therapeutic FTY720 could reverse the receptor maladaptation. A significant increase in S1P1R was detected in synaptosomal lysates from FTY720-untreated EAE mice, which largely reversed in EAE mice treated with FTY720 (Figure 8A,B). Similarly, the density of S1P3Rs in synaptosomal lysates (Figure 8C,D) from FTY720-untreated EAE mice was higher than that in control animals (Figure 8C) but diminished in FTY720-treated EAE mice (Figure 8D). 

Based on these results, we asked whether the changes in receptor densities in EAE mice cortical synaptosomes could be paralleled by a re-distribution of S1P1/3R in plasma membranes. To answer this question, biotinylation studies were carried out using synaptosomal lysates from the cortex of control and EAE mice. Again, S1P1R and S1P3R were detected in synaptosomal lysates from control and FTY720-untreated EAE mice, but not in biotinylated samples (S1P1R: Figure 8E; S1P3R: Figure 8F). GluA2, here used as a positive biotinylation control, was observed in both synaptosomal lysates and biotinylated samples (Figure 8E,F).

**Figure 9:**
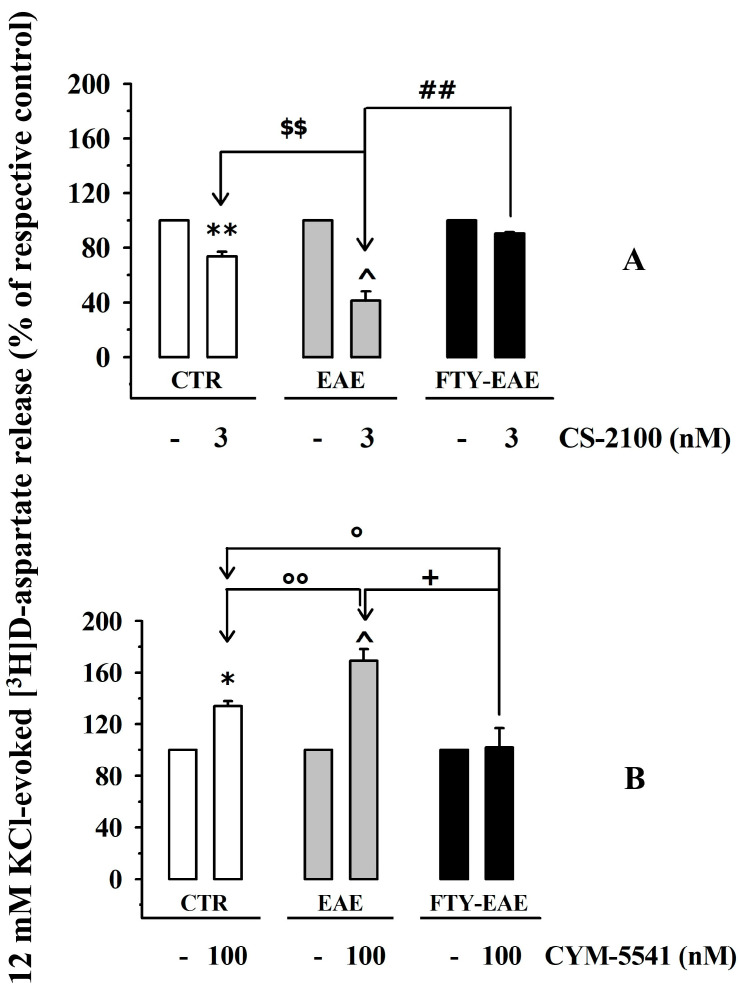
Effects of CS-2100 and CYM-5541 on the 12 mM KCl-induced [^3^H]D-aspartate in cortical synaptosomes of EAE mice: impact of FTY720 treatment. Cortical synaptosomes from control (white bar), FTY720-untreated EAE (grey bar), and FTY720-treated EAE (black bar) mice were exposed in superfusion to 12 mM KCl in the absence or the presence of CS-2100 (3 nM) (**A**) or CYM-5541 (100 nM) (**B**) and the ligand-induced changes in [^3^H]D-aspartate exocytosis were expressed as a percentage of the respective control (12 mM KCl-induced [^3^H]D-aspartate release). Data are expressed as mean ± SEM of at least five experiments run in triplicate. * *p* < 0.05 vs. 12 mM KCl-evoked tritium release from control synaptosomes; ** *p* < 0.01 vs. 12 mM KCl-evoked tritium release from control synaptosomes; $$ *p* < 0.01 vs. 12 mM KCl/3 nM CS-2100-evoked tritium release from control synaptosomes; ° *p* < 0.05 vs. 12 mM KCl/100 nM CYM-5541-evoked tritium release from control synaptosomes; °° *p* < 0.01 vs. 12 mM KCl/100 nM CYM-5541-evoked tritium release from control synaptosomes; ^ *p* < 0.05 vs. 12 mM KCl-evoked tritium release from EAE synaptosomes; ## *p* < 0.01 vs. 12 mM KCl/3 nM CS-2100-evoked tritium release from EAE synaptosomes; ^+^ *p* < 0.05 vs. 12 mM KCl/100 nM CYM-5541-evoked tritium release from EAE synaptosomes.

Finally, we quantified the impact of CS-2100 (3 nM, Figure 9A) and CYM-5541 (100 nM, Figure 9B) on the 12 mM KCl-evoked release of [^3^H]D-aspartate from cortical synaptosomes of control, FTY720-untreated EAE mice, and FTY720-treated EAE mice.

The inhibitory effect of CS-2100 on the KCl-evoked release of [^3^H]D-aspartate was amplified in cortical synaptosomes of FTY720-untreated EAE mice but largely recovered in synaptosomes of FTY720-treated EAE mice (Figure 9A), consistent with the changes in S1P1R densities described in Figure 8A,B. Similarly, the release-promoting activity of CYM-5541 (100 nM) in cortical synaptosomes of FTY720-untreated EAE mice was improved, but significantly reduced in cortical synaptosomes isolated from FTY720-treated EAE mice (Figure 9B), in line with the changes in receptor densities described in Figure 8C,D. 

## 4. Discussion

In recent years, accumulated data has proven that, in EAE mice, S1PRs ligands (i.e., FTY720 and siponimod) modulate neuroprotective glutamatergic transmission [16,17,35]. In general, the beneficial activity was ascribed to “immunological” effects elicited by S1PRs in immune cells [11]. However, since S1PR modulators can enter the CNS [36] and activate S1PRs located on astrocytes and neurons, the possibility that these drugs may also elicit neuroprotective “non immunological” events is attractive and deserves attention [4,16,37]. “in vitro” FTY720, for istance, can either increase [38] or decrease [21] glutamate exocytosis. 

FTY720-mediated inhibition of glutamate overflow from cortical synaptosomes has been proposed to involve subtype 1 presynaptic release-regulating S1P receptors. The conclusion relies on the finding that the inhibitory effect elicited by this drug is mimicked by the S1P1R agonist, SEW2871 [21,22], and was partially reverted by the S1P1R antagonist, W146. The latter compound, however, imitated FTY720 when tested alone [21], shedding some doubt on the pharmacological characterization of the receptor subtype involved in the inhibitory effect. The functional observations were also supported by biochemical results that showed the presence of S1P1R proteins in cortical glutamatergic synaptosomes [21,22]. 

On the other hand, the receptor subtype involved in FTY720-mediated glutamate release has not been characterized, but S1P3R has been proposed as a suitable candidate. The hypothesis relies on the observation that the newly synthesized synaptic S1P acting on synaptic S1P3Rs were shown to reorganize the STX1A distribution in presynaptic terminals [14,20], an event that could favor glutamate exocytosis [14,23]. Although attractive, this hypothesis was excluded by Wang and colleagues [21] based on the findings that (i) suramin (an S1P3/S1P5 receptor antagonist) did not modify the FTY720-mediated inhibition of glutamate exocytosis from cortical synaptosomes and (ii) S1P3R immunostaining was not detected in these particles. However, Skoug and colleagues [22] later highlighted a clear S1P3R immunopositivity in cortical synaptosomes, which we confirmed in the present study, reproposing the question of the existence of presynaptic release-regulating S1P3Rs in glutamatergic cortical nerve endings.

The first aim of our study was to provide new functional and biochemical evidence that could improve the pharmacological characterization of presynaptic inhibitory S1P1Rs in cortical glutamatergic synaptosomes and support the existence of presynaptic facilitatory S1P3Rs in these particles. To this aim, we first carried out release studies using an experimental technique set up in our laboratory several years ago: the “superfusion of a thin layer of synaptosomes”. This technique is widely recognized as an appropriate approach for demonstrating the existence of presynaptic release-regulating receptors and studying their mechanism of control of transmitter release [27]. Its main feature is the continuous up–down superfusion that minimizes the autocrine/paracrine effects elicited by endogenous compounds (which are rapidly removed), preserving the probability that ligand(s) exogenously added to the superfusion medium can access and activate presynaptic receptors [27,39,40,41,42]. 

Under these experimental conditions, the findings that the KCl-evoked [^3^H]D-aspartate overflow is significantly modified by S1P1R or S1P3R agonists concomitantly added to the depolarizing stimulus (i.e., CS-2100 reduced tritium overflow while CYM-5541 increased it) are consistent with the presence of inhibitory presynaptic S1P1Rs and facilitatory presynaptic S1P3Rs in superfused cortical synaptosomes. The conclusion is further strengthened by the results obtained using the S1P1R antagonist, Ex 26, and the S1P3R antagonist, TY-52156. The two antagonists, unable to modify the tritium overflow on their own, prevented the presynaptic control of glutamate exocytosis mediated by CS-2100 and CYM-5541, respectively. These observations confirm—from a functional point of view—the existence of presynaptic release-regulating S1P1R and S1P3R in mice cortical glutamatergic synaptosomes. 

The functional observations are supported by biochemical data from Western blot analysis and confocal microscopy, which highlighted the presence of S1P1Rs and S1P3R proteins in cortical glutamatergic synaptosomes [21,22]. Besides confirming the presence of S1P1R proteins in cortical synaptosomes [21], the biochemical observations unveiled clear S1P3R immunopositivity in the synaptosomal particles (see also Skoug and colleagues [22]), specifically in glutamatergic nerve terminals (VGLUT1-positive particles) specialized for exocytosis (STX1A-positive particles). 

Release experiments were used also to investigate the impact of the endogenous S1PR ligand, S1P, on glutamate release. The results highlighted a lack of efficacy of the drug in controlling the tritium overflow. S1P (up to 1 nM) did not modify the spontaneous release of tritium, and only slightly, but not significantly, reduced KCl-evoked tritium exocytosis. Several events might explain SIP’s ineffectiveness. First, the low SIP efficiency may be apparent, representing the sum of opposite effects elicited by the two presynaptic S1P1 and S1P3 receptors, that, if activated concomitantly, can compensate for each other. Alternatively, its low efficiency in modulating glutamate exocytosis might be associated with its instability, since this compound is metabolically degraded in the presence of biological membranes in vitro [43]. Lastly, transient depolarization [14] may increase the endogenous content of S1P within synaptosomes (cortical synaptosomes possess SphK [14,21]) to a level sufficient to activate and even almost saturate the S1PRs, which responses cannot be further increased by exogenous S1P. The last hypothesis is attractive because it is indirectly supported by the finding that the 12 mM KCl-evoked release of [^3^H]D-aspartate in the presence of the competitive SphK inhibitor DMS (which prevents endogenous production of S1P) is significantly lower than the tritium overflow in the absence of the enzyme inhibitor (see also [19]). Furthermore,, a possible role of endogenous S1P in priming glutamate exocytosis is indirectly suggested by the responsiveness of synaptosomes to CYM-5541. The compound is an allosteric S1P3R modulator and therefore requires the presence of an orthosteric ligand to exert its activity. Its efficiency in potentiating glutamate exocytosis indirectly implies the presence of an endogenous orthosteric agonist (i.e., S1P) that would activate S1P3Rs and permit their allosteric modulation. If this is the case, the S1P3R antagonist, TY-52156, would be expected to compete with endogenous S1P for S1P3Rs, hence reducing glutamate exocytosis. Contrary to expectation, the antagonist was inactive, suggesting that further studies are needed to address this issue.

Another unexpected observation concerns the distribution of S1PRs in synaptosomes. It is proposed that S1PRs are located on cellular plasma membranes and undergo a rapid internalization upon activation (the functional antagonism [7,11,44]). Based on this assumption, biotinylation studies were carried out under resting conditions (to limit the endogenous production of S1P, which can indirectly modify receptor trafficking) and in the absence of exogenous S1PR ligands to verify the presence of S1PRs in the plasma membranes of synaptosomes. Surprisingly, biotinylated samples did not show clear immunopositivity for both receptors, suggesting that, if present, the proteins were largely “inaccessible” to biotin. A plausible explanation could be that a large part of the S1PRs preferentially localize on the inner side of the membranes or even in the cytosolic pools. Accordingly, Skoug et al. [22] demonstrated that, in cortical synaptosomes, S1P1/3 receptors were scarcely present in the pre-and post-synaptic components of the synaptic active zone but were concentrated in the remaining parts of synaptosomal plasma membranes, namely the “extrasynaptic” component, i.e., those parts of plasma membranes outside the synaptic active zone or of cytosolic structures (see also [45]). If this is the case, S1PR agonists would access the receptors by entering synaptosomes (indeed, the partition coefficient of the S1P1R and the S1P3R ligands would allow their diffusion across the plasma membrane) to modulate glutamate exocytosis. An alternative hypothesis is that the transient depolarization of synaptosomes may favor a rapid redistribution of the receptors (possibly sustained by the concomitant production of endogenous S1P in isolated nerve terminals), which could promote the interaction with ligands. Whatever the mechanism, it seems conceivable to conclude that the trafficking of S1PRs on plasma membranes is an event that is more complex than expected.

The second aim of this study was to verify whether, and to what extent, ongoing neuroinflammatory and immune events can affect the density, distribution, and role of the presynaptic release-regulating S1P1 and S1P3 receptors on glutamatergic nerve endings. To answer this question, we focused on EAE mice, animal models that recapitulate several main inflammatory and immune responses affecting MS patients. The progression of MS and EAE relies largely on the pathogenetic activation of astrocytes, microglia, and infiltrating lymphocytes, which contribute to the maladaptive events subserving the gravity of the disease. It is proposed that changes in the expression/function of the central S1PRs, including those controlling glutamate exocytosis, would participate in the progression of neurological symptoms [8,46,47]. Accordingly, FTY720 treatment efficiently reversed these clinical signs, reducing the gravity of the disease [6,7,11,16,17].

In this context, besides highlighting the possible biochemical and functional changes of S1P1Rs and S1P3Rs in cortical EAE synaptosomes, the study aimed to evaluate the impact of the administration of therapeutic FTY720 on these receptors and their efficiency in controlling glutamate exocytosis. The working hypothesis is based on evidence in the literature demonstrating that the compound recovers central glutamatergic derangements [6,16,17] and modulates the expression of S1PR mRNA [48,49] in these mice. 

The nerve terminals isolated from the cortex of EAE mice showed an increased density of the two S1PRs. The EAE-induced adaptation, however, did not imply the redistribution of the receptor proteins in the synaptosomal plasma membranes. Rather, biotinylation studies confirmed that S1P1 and S1P3 receptor proteins were still largely inaccessible to biotin. Interestingly, the chronic administration of therapeutic FTY720 significantly reverted the S1PR densities to physiological levels, unaffecting their preferential internal distribution. 

We also observed a positive correlation between biochemical observations and functional results. The overexpression of both receptors in cortical synaptosomal lysates from untreated EAE mice was paralleled by changes in S1P1R and S1P3R efficiency in controlling glutamate exocytosis. Thus, FTY720 administration, which significantly reduced the densities of both S1P1 and S1P3 receptors, also restored their release-regulating activity to levels consistent with those detected in control animals.

In conclusion, the findings obtained in the first part of the study demonstrate that mouse cortical glutamatergic nerve endings are endowed with presynaptic release-regulating S1P1 and S1P3 receptors. The two receptors are inactive under resting conditions, but control glutamate exocytosis elicited by mild depolarizing stimuli, with opposite outcomes; S1P1R inhibits glutamate exocytosis while S1P3R potentiates it. Our data does not allow us to speculate on whether the two receptors colocalize on the same terminals or if they are expressed in different structures; however, it suggests a preferential distribution of both receptor proteins on the inner side of the synaptosomal particles. Furthermore, our study allows some hypotheses on the role of endogenous S1P, including its overexpression and accumulation in nerve terminals following exposure to a depolarizing stimulus. 

The second part of the study focused on EAE mice and on the efficacy of FTY720 treatment in recovering synaptic maladaptation that could involve S1P1 and S1P3 receptors controlling glutamate exocytosis. We provided evidence demonstrating that both receptors undergo changes in terms of protein density and efficiency when controlling glutamate exocytosis at the acute stage of EAE. Interestingly, these EAE-related adaptations largely recover following therapeutic administration of FTY720. To note, the preferential inner distribution of both receptor proteins is unaltered in EAE mice and largely unmodified by FTY720 treatment. 

Our data provides knowledge on the impact of FTY720 on S1PR-mediated control of glutamatergic synaptic transmission in the cortex of mammals and unveils presynaptic adaptations that could play an important role in the synaptic de-synchronization that typify the course of MS. 

Our research was limited to the cortex and EAE mice. It will be important in the future to extend the research to study the existence and functional role of S1P receptors (including the S1P2 and S1P5 receptor subtypes) as presynaptic modulators of chemical transmission in other regions of the CNS to deepen our knowledge of the role of S1P in the control of synaptic plasticity. The results of these studies will support the use of FTY720 and related drugs in the management of central disorders other than MS.

## Data Availability

Not applicable.
